# Evaluating the Impact of Catheter Ablation on Cardiovascular and Cerebral Outcomes in Atrial Fibrillation With Heart Failure and Preserved Ejection Fraction

**DOI:** 10.1002/clc.70220

**Published:** 2025-11-07

**Authors:** Wei‐Chieh Lee, Wan‐Hsuan Hsu, Chih‐Cheng Lai, Wei‐Ting Chang, Chia‐Te Liao, Jhih‐Yuan Shih, Zhih‐Cherng Chen, Hsiu‐Yu Fang, Mien‐Cheng Chen

**Affiliations:** ^1^ School of Medicine, College of Medicine National Sun Yat‐sen University Kaohsiung Taiwan; ^2^ Department of Internal Medicine Division of Cardiology, Chi Mei Medical Center Tainan Taiwan; ^3^ Department of Internal Medicine Chi Mei Medical Center Tainan Taiwan; ^4^ Department of Internal Medicine, Division of Cardiology, Kaohsiung Chang Gung Memorial Hospital Chang Gung University College of Medicine Kaohsiung Taiwan; ^5^ Department of Internal Medicine Division of Cardiology, Jen‐Ai Hospital Taichung Taiwan

**Keywords:** atrial fibrillation, catheter ablation, heart failure with acute exacerbation, heart failure with preserved ejection fraction, ischemic stroke

## Abstract

**Background:**

Evidence supporting catheter ablation (CA) for atrial fibrillation (AF) in heart failure with preserved ejection fraction (HFpEF) is limited. This study evaluated the impact of CA on clinical outcomes in patients with AF and HFpEF using a global clinical database.

**Methods:**

The TriNetX research network identified patients aged ≥ 18 years with AF and HFpEF (February 2014 to June 2024). Patients were categorized by whether they underwent CA for AF. Primary outcomes included all‐cause mortality, heart failure (HF) with acute exacerbation, and ischemic stroke. Secondary outcomes included progression to mildly reduced or reduced ejection fraction (EF) during follow‐up.

**Results:**

Patients receiving CA showed lower incidences of all‐cause mortality, HF exacerbation, and ischemic stroke. There was a trend of less patients with progression to reduced EF in patients with CA. The reduction in mortality was consistent across all subgroups, while stroke reduction was more significant in females, those with better EF, without chronic kidney disease (CKD) or diabetes mellitus (DM), with hypertension (HTN), and with paroxysmal AF. The benefits in reducing HF exacerbation were particularly notable in females, those with better EF, without CKD, and with HTN.

**Conclusions:**

In patients with AF and HFpEF, CA provided cardiovascular and cerebral benefits and might reduce the risk of progression to HFrEF over 5 years of follow‐up. Additionally, CA was associated with a reduction in all‐cause mortality in patients with AF and HFpEF.

## Graphical abstract

This study summarizes the 5‐year outcomes of catheter ablation in patients with atrial fibrillation and HFpEF. Compared to medical therapy alone, ablation was associated with reduced all‐cause mortality, ischemic stroke, and heart failure exacerbation, based on matched cohort analysis from a global real‐world database.

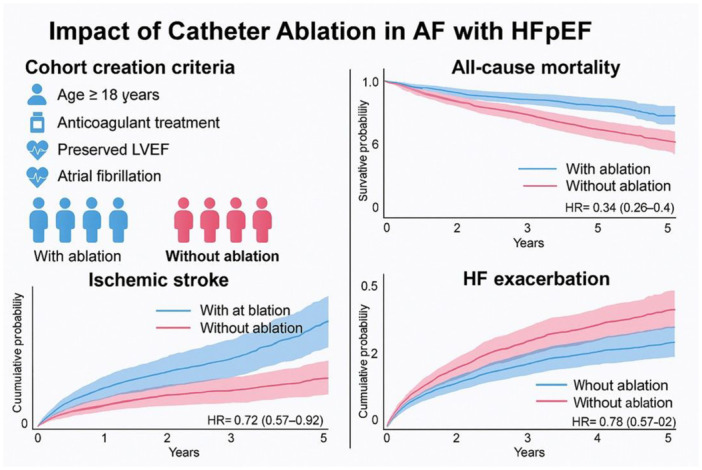

## 1 Background

Due to an aging population, the prevalence of atrial fibrillation (AF) is increasing, and recent evidence of cardiovascular and cerebral benefits of early rhythm control strategies, making catheter ablation (CA) a reasonable method for rhythm control, especially at the earlier stages of AF [1, 2, 3]. Landmark trials have demonstrated the safety and efficacy of CA in patients with AF and heart failure (HF) [4, 5, 6]. Moreover, CA has been shown to reduce the progression to persistent AF [7]. As a result, there is growing interest in shifting from rate control approaches to rhythm control strategies earlier in the disease course of AF. In the population with AF and end‐stage HF, the combination of CA and guideline‐directed medical therapy was associated with a lower likelihood of a composite of all‐cause mortality, implantation of a left ventricular assist device, or urgent heart transplantation than medical therapy alone [6]. Furthermore, CA also provides the largest improvement in left ventricular ejection fraction (LVEF) in patients with HF with reduced ejection fraction (HFrEF) [8]. In a small population study, CA improves invasive exercise hemodynamic parameters, exercise capacity, and quality of life in patients with AF and heart failure with preserved ejection fraction (HFpEF) [9, 10]. However, a meta‐analysis found that CA had limited or no benefit in terms of HF events and cardiovascular mortality for patients with AF and HFpEF [11]. Therefore, the clinical benefits of CA in terms of cardiovascular and cerebral outcomes appear to be controversial and inconsistent in patients with AF and HFpEF. Accordingly, we aimed to explore the effects of CA on clinical outcomes in patients with AF and HFpEF by using a real‐world and global federated health network.

## 2 Methods

### 2.1 Patient Population and Data Source

The study design and participant selection process for a global collaborative network analysis conducted within the TriNetX platform, which includes data from 120 healthcare organizations (HCOs) worldwide (Figure S1). This study retrieved data on June 24, 2024, from a total of 128 873 996 patients. From this population, 65 160 687 patients aged 18 and older who visited HCOs three or more times since February 1, 2014, were identified. Among these, 32 024 patients were diagnosed with HFpEF and AF. The associated diagnosis codes were listed in Supporting Information S1: Table 1.

The exclusion criteria applied were as follows: (1) Patients who did not receive anticoagulant treatment (*n* = 14 284), (2) patients who ever received heart valve replacement (*n* = 753), (3) patients diagnosed with chronic kidney disease (CKD) stage 5 or end‐stage renal disease (ESRD) (*n* = 894), and (4) patients diagnosed with supraventricular tachycardia (SVT) or atrial flutter alone (*n* = 2020).

After applying these criteria, 1234 patients received CA for AF, while 13 500 patients did not receive CA. Propensity score matching (PSM) (1:1) was performed based on age, gender, race, ethnicity, comorbid conditions, medications, and laboratory features (Supporting Information S1: Table 2). The index date for the ablation group was defined as the first date of receiving CA, and the index date for the without ablation group was defined as the first date of co‐occurrent diagnosis of AF and HFpEF. This matching process resulted in two groups: 1152 patients in the ablation group and 1152 patients in the without ablation group.

### 2.2 Ethical Statement

The authors analyzed and interpreted the data, and all authors reviewed the manuscript to confirm the accuracy and completeness of the information. The protocol was exempt from institutional review board approval by the Chi Mei Medical Center, as aggregate deidentified data from a research network database were used. The study findings are reported in accordance with the Strengthening the Reporting of Observational Studies in Epidemiology (STROBE) guidelines for cohort studies.

### 2.3 Study Outcomes

The primary composite outcome of this study included HF with acute exacerbation (defined by International Classification of Diseases [ICD] codes or the need for intravenous diuretics), ischemic stroke, and all‐cause mortality (Supporting Information S1: Table 3). Follow‐up LVEF values used to support the diagnosis of reduced or mildly reduced ejection fraction, when available, were recorded after the appearance of new diagnostic codes. Outcomes were analyzed over a period of 3 months to 5 years following the index AF ablation to account for a 3‐month blanking period after the index event in the ablation population and after the diagnosis of AF and HFpEF in the without ablation population.

### 2.4 Statistical Analysis

The patient population was stratified into two cohorts: Those with ablation and those without ablation. Continuous variables are presented as mean ± standard difference (SD) and were compared between the cohorts using independent‐sample *t*‐tests. Categorical variables are reported as numbers (percentages) and were compared using the chi‐squared test. To control baseline differences between the cohorts, 1:1 PSM was performed using a built‐in PSM algorithm, which employs the greedy nearest‐neighbor method with a caliper of 0.1 pooled SDs. Any characteristic with a standardized mean difference of < 0.1 between cohorts was considered well‐matched. Adjusted hazard ratios (HRs) with 95% confidence intervals (CIs) were calculated for outcomes. Survival analysis was conducted by plotting Kaplan−Meier curves and using log‐rank tests to compare the two cohorts. Statistical significance was set at a two‐sided *p* value of < 0.05. Post hoc statistical power for each outcome was calculated using the Schoenfeld method, based on observed event counts and effect sizes, with a two‐sided *α* level of 0.05. Subgroup analyses were undertaken based on age, sex, LVEF, presence of coexisting comorbid conditions, including CKD, hypertension (HTN), type 2 diabetes mellitus (DM), and dyslipidemia, and types of AF (Supporting Information S1: Table 4). All statistical analyses were performed using the TriNetX online platform and Stata 18.0 for statistical computing. To evaluate the impact of missing data and confirm adequate study power, we performed post hoc power calculations for each outcome using the Schoenfeld method, based on observed events and effect sizes with a two‐sided *α* of 0.05 (Supporting Information S1: Table 5).

## 3 Results

### 3.1 Comparison of Baseline Characteristics Between Patients With Ablation or Without Ablation

The baseline characteristics of patients in the ablation and without ablation groups were listed in Table 1 and revealed significant disparities before PSM. Before PSM, the ablation group had a younger age (67.7 ± 9.7 years) compared to the without ablation group (73.1 ± 11.8 years), with a standardized difference of 0.500. There were also notable differences in the prevalence of paroxysmal AF, which was significantly higher in the ablation group (76.6% vs. 27.8%, standardized difference of 1.119). Additionally, the use of β‐blockers (79.9% in the ablation group vs. 61.2% in the without ablation group, standardized difference of 0.419) and antiarrhythmic drugs (74.5% vs. 54.2%, standardized difference of 0.434) was higher in the ablation group.

**Table 1:** Baseline characteristics in with ablation and without ablation groups before and after propensity score matching.

	Before PSM			After PSM			
	With ablation (*n* = 1234)	Without abaltion (*n* = 13 500)	Std diff.	With ablation (*n* = 1152)	Without abaltion (*n* = 1152)	Std diff.
Demographics						
*Age at index (years old)*	67.7 ± 9.7	73.1 ± 11.8	0.500	68.3 ± 9.2	68.1 ± 13.2	0.019
*Sex*						
Male	660 (53.50)	6784 (50.30)	0.065	605 (52.50)	614 (53.30)	0.016
Female	571 (46.30)	6667 (49.40)	0.062	545 (47.30)	535 (46.40)	0.017
*Race*						
White	1106 (89.60)	11 548 (85.50)	0.124	1033 (89.70)	1036 (89.90)	0.009
Black or African American	55 (4.50)	915 (6.80)	0.101	53 (4.60)	47 (4.10)	0.026
Asian	18 (1.50)	219 (1.60)	0.013	17 (1.50)	15 (1.30)	0.015
Native Hawaiian or Other Pacific Islander	10 (0.80)	10 (0.10)	0.111	10 (0.90)	10 (0.90)	< 0.001
American Indian or Alaska Native	10 (0.80)	23 (0.20)	0.092	10 (0.90)	10 (0.90)	< 0.001
Other Race	20 (1.60)	364 (2.70)	0.074	18 (1.60)	20 (1.70)	0.014
Unknown Race	30 (2.40)	421 (3.10)	0.042	26 (2.30)	29 (2.50)	0.017
*Ethnicity*						
Not Hispanic or Latino	1158 (93.80)	12 568 (93.10)	0.030	1087 (94.40)	1081 (93.80)	0.022
Hispanic or Latino	45 (3.60)	429 (3.20)	0.026	39 (3.40)	40 (3.50)	0.005
Unknown Ethnicity	31 (2.50)	503 (3.70)	0.070	26 (2.30)	31 (2.70)	0.028
Comorbid conditions						
*Cardiovascular diseases*						
Hypertensive diseases	1022 (82.80)	9483 (70.20)	0.300	956 (83.00)	943 (81.90)	0.030
Ischemic heart diseases	610 (49.40)	6988 (51.80)	0.047	577 (50.10)	588 (51.00)	0.019
Acute diastolic heart failure	150 (12.20)	849 (6.30)	0.204	133 (11.50)	118 (10.20)	0.042
Paroxysmal atrial fibrillation	945 (76.60)	3755 (27.80)	1.119	863 (74.90)	858 (74.50)	0.010
*Metabolic disorders*						
Dyslipidemia	828 (67.10)	7561 (56.00)	0.229	779 (67.60)	752 (65.30)	0.050
Overweight and obesity	566 (45.90)	3569 (26.40)	0.413	511 (44.40)	525 (45.60)	0.024
Type 2 diabetes mellitus	456 (37.00)	4527 (33.50)	0.072	426 (37.00)	434 (37.70)	0.014
Disorders of thyroid gland	365 (29.60)	3146 (23.30)	0.143	343 (29.80)	345 (29.90)	0.004
*Chronic kidney diseases*						
Stage 3	139 (11.30)	2089 (15.50)	0.124	135 (11.70)	136 (11.80)	0.003
Stage 4	26 (2.10)	533 (3.90)	0.108	25 (2.20)	32 (2.80)	0.039
Cerebral infarction	183 (14.80)	2206 (16.30)	0.042	178 (15.40)	190 (16.50)	0.028
Medications						
β‐blockers	986 (79.90)	8261 (61.20)	0.419	908 (78.80)	902 (78.30)	0.013
Antiarrhythmic	919 (74.50)	7312 (54.20)	0.434	848 (73.60)	840 (72.90)	0.016
Diuretics	804 (65.20)	7980 (59.10)	0.125	749 (65.00)	718 (62.30)	0.056
Statin	651 (52.80)	6806 (50.40)	0.047	613 (53.20)	593 (51.50)	0.035
Antiplatelet	558 (45.20)	7130 (52.80)	0.152	545 (47.30)	541 (47.00)	0.007
ARBs	372 (30.10)	2490 (18.40)	0.275	325 (28.20)	351 (30.50)	0.050
ACE inhibitors	342 (27.70)	3284 (24.30)	0.077	321 (27.90)	323 (28.00)	0.004
Insulin	337 (27.30)	4365 (32.30)	0.110	325 (28.20)	329 (28.60)	0.008
Digitalis	170 (13.80)	781 (5.80)	0.271	152 (13.20)	151 (13.10)	0.003
Metformin	153 (12.40)	1384 (10.30)	0.068	145 (12.60)	148 (12.80)	0.008
SGLT2 inhibitors	78 (6.30)	265 (2.00)	0.220	59 (5.10)	53 (4.60)	0.024
Glipizide	38 (3.10)	442 (3.30)	0.011	36 (3.10)	38 (3.30)	0.010
Laboratory values						
Creatinine (mg/dL)	1.1 ± 0.4	1.2 ± 1.3	0.148	1.1 ± 0.5	1.1 ± 0.6	0.016
LDL (mg/dL)	86.0 ± 36.0	82.2 ± 35.7	0.105	85.9 ± 35.8	82.5 ± 34.0	0.097
HbA1c ≥ 7%	140 (11.30)	1749 (13.00)	0.049	134 (11.60)	132 (11.50)	0.005
BNP ≥ 150 pg/mL	295 (23.90)	2376 (17.60)	0.156	265 (23.00)	269 (23.40)	0.008
NT‐proBNP ≥ 450 pg/mL	296 (24.00)	2787 (20.60)	0.080	279 (24.20)	270 (23.40)	0.018
LVEF (%)	58.9 ± 6.5	59.8 ± 9.5	0.119	58.8 ± 6.5	59.3 ± 10.2	0.062

*Note:* Values are mean ± standard deviation or number (%). Any characteristic with a standard difference between cohorts < 0.10 was considered to be well matched.

Abbreviations: ACEIs, angiotensin‐converting enzyme inhibitors; ARBs, angiotensin receptor blockers; BNP, B‐type natriuretic peptide; HMG‐CoA, hydroxymethylglutaryl‐CoA; LDL‐C, low‐density lipoprotein‐cholesterol; PSM, propensity score matching; SD, standardized difference; SGLT2, sodium‐glucose co‐transporter 2.

After PSM, these differences in the baseline characteristics were substantially minimized, resulting in a more balanced in the baseline characteristics between the two groups. For example, the average age was nearly equalized (68.3 ± 9.2 years in the ablation group vs. 68.1 ± 13.2 years in the without ablation group, standardized difference of 0.019). The prevalence of paroxysmal AF also became comparable (74.9% in the ablation group vs. 74.5% in the without ablation group, standardized difference of 0.010). The use of β‐blockers (78.8% vs. 78.3%, standardized difference of 0.013) and antiarrhythmic drugs (73.6% vs. 72.9%, standardized difference of 0.016) was balanced as well. These adjustments indicate that PSM effectively reduced the baseline disparities between the ablation and without ablation groups, ensuring that the subsequent analyses of clinical outcomes are more robust and reliable. The significant differences observed by pre‐PSM highlight the inherent variabilities in patient characteristics, while the post‐PSM balance underscores the successful application of matching techniques to create equivalent comparison groups.

### 3.2 Comparison of Clinical Outcomes Between Patients With and Without Ablation After PSM

The median follow‐up duration, beginning 90 days after the index date, was 773 days (interquartile range [IQR], 91–1151) in the with ablation group and 627 days (IQR, 91–1131) in the without ablation group. The HRs and incidences of clinical outcomes between patients with and without ablation during a 5‐year follow‐up period after PSM are listed in Table 2. Notably, patients who underwent ablation had significantly lower all‐cause mortality (6.1% vs. 16.9%, HR: 0.34, 95% CI: 0.26−0.44, *p* < 0.0001) and reduced incidence of ischemic stroke (10.1% vs. 12.8%, HR: 0.72, 95% CI: 0.57−0.92, *p* = 0.0080). Additionally, HF with acute exacerbation was significantly less frequent in the ablation group (22.7% vs. 27.0%, HR: 0.78, 95% CI: 0.67−0.92, *p* = 0.0035). Moreover, there was a trend of less patients with progression to reduced EF in patients with CA.

**Table 2:** The hazard ratios and incidences for comparing matched with ablation and without ablation group during the 5‐year follow‐up period.

	*N* (%) of patients with outcome		HR (95% CI)	Log‐rank *p* value
	With ablation (*n* = 1152)	Without ablation (*n* = 1152)		
All‐cause mortality (%)	70 (6.1)	195 (16.9)	0.34 (0.26, 0.44)	< 0.0001
HF exacerbation (%)	262 (22.7)	311 (27.0)	0.78 (0.67, 0.92)	0.0035
LVEF progress to mildly reduced EF (%)	129 (11.2)	123 (10.7)	1.02 (0.79, 1.30)	0.8926
LVEF progress to reduced EF (%)	57 (4.9)	75 (6.5)	0.72 (0.51, 1.02)	0.0634
Ischemic stroke (%)	116 (10.1)	148 (12.8)	0.72 (0.57, 0.92)	0.0080

*Note:* Data are expressed as number (percentage).

Abbreviations: CI, confidence interval; HF, heart failure; HR, hazard ratio; LVEF, left ventricular ejection fraction.

### 3.3 Kaplan−Meier Curve Analysis of the HF With Acute Exacerbation, Ischemic Stroke, and All‐Cause Mortality Between Patients With and Without Ablation After PSM

**Figure 1:**
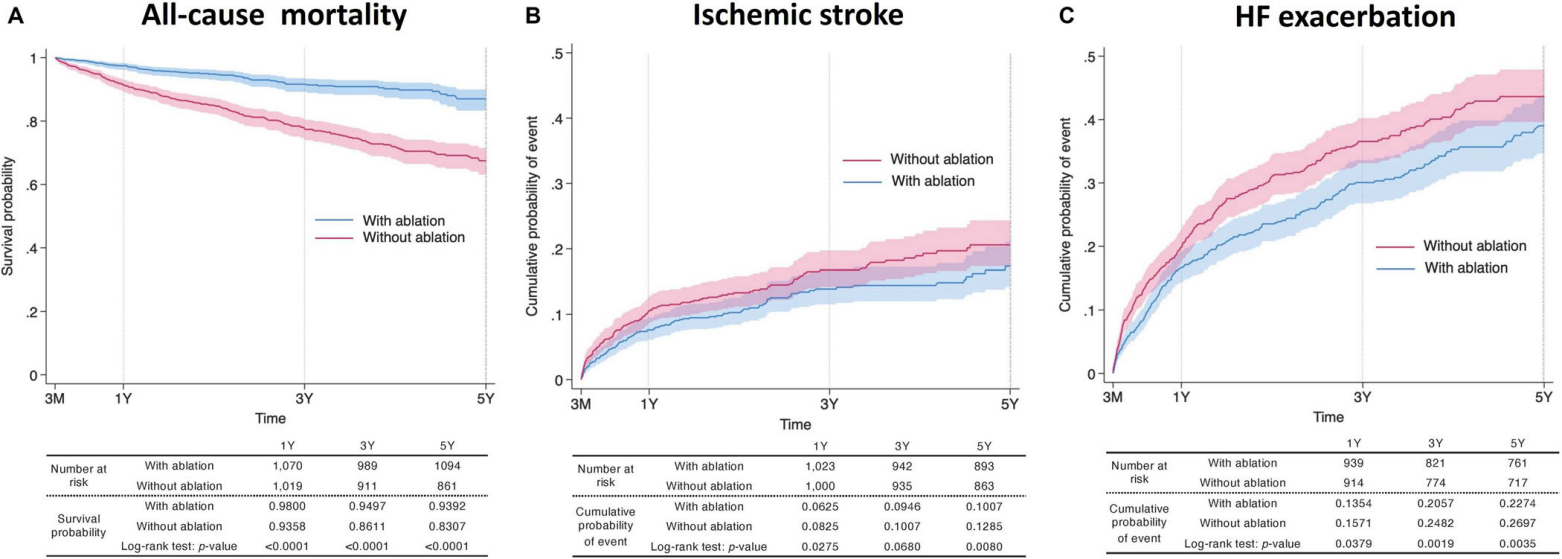
Kaplan−Meier curves for all‐cause mortality, stroke, and HF exacerbation. (A) Kaplan−Meier survival curves for all‐cause mortality among patients with HFpEF and AF, comparing those who received ablation therapy (blue line) and those who did not (red line). Survival probability at 1 year, 3 years, and 5 years was higher in the ablation group compared to the non‐ablation group. The log‐rank test indicated a significant difference (*p* < 0.0001) between the two groups. (B) Cumulative probability of ischemic stroke over time for patients with HFpEF and AF, comparing those who received ablation therapy (blue line) and those who did not (red line). The cumulative probability of stroke was lower in the ablation group at 1 year, 3 years, and 5 years. The log‐rank test showed a significant difference (*p* = 0.0080). (C) Cumulative probability of HF with acute exacerbation over time for patients with HFpEF and AF, comparing those who received ablation therapy (blue line) and those who did not (red line). The cumulative probability of worsening of HF was lower in the ablation group at 1 year, 3 years, and 5 years. The log‐rank test showed a significant difference (*p* = 0.0001). Each panel includes shaded areas representing the 95% confidence intervals for the survival and event probability estimates. AF, atrial fibrillation; HF, heart failure; HFpEF, heart failure with preserved ejection fraction.

Three Kaplan−Meier curves comparing outcomes between patients with and without ablation presented in Figure 1. The survival probability for all‐cause mortality over 5 years demonstrated higher survival rates in patients with ablation, with a significant log‐rank *p*‐value of < 0.0001 (Figure 1A). Specifically, the 1‐year survival probability is 0.9800 for those with ablation and 0.9358 for those without ablation. The cumulative probability of stroke over 5 years demonstrated a lower stroke rate in patients with ablation, with a significant log‐rank *p*‐value of 0.0080 (Figure 1B). The 1‐year cumulative probability is 0.0625 for those with ablation compared to 0.0825 for those without ablation (*p*‐value, 0.0275). The cumulative probability of HF with acute exacerbation over 5 years, with patients who underwent ablation experiencing fewer events, marked by a significant log‐rank *p*‐value of 0.0035 (Figure 1C). The 1‐year cumulative probability of HF with acute exacerbation is 0.1354 for patients with ablation and 0.1571 for those without ablation (*p*‐value, 0.0379). Overall, these figures indicate the beneficial impact of ablation on reducing mortality, stroke, and HF with acute exacerbation in patients with AF.

### 3.4 Subgroup Analysis of HF With Acute Exacerbation, Ischemic Stroke, and All‐Cause Mortality Between Patients With and Without Ablation

**Figure 2:**
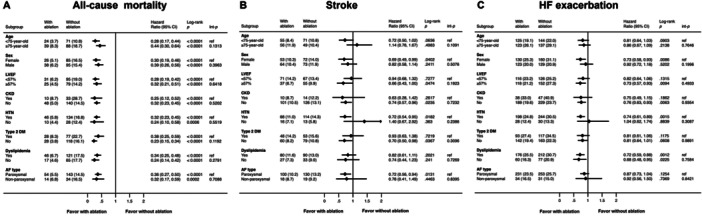
Subgroup analysis of HF with acute exacerbation, ischemic stroke, and all‐cause mortality between patients with and without ablation. (A) Subgroup analysis of hazard ratios for all‐cause mortality between patients with ablation versus without ablation. The HRs indicate a significantly lower risk of all‐cause mortality for patients who received ablation therapy across all subgroups, with all log‐rank *p*‐values being < 0.001, indicating high statistical significance. (B) Subgroup analysis of hazard ratios for ischemic stroke between patients with ablation versus without ablation. The HRs indicate a varying degree of risk for ischemic stroke across different subgroups, with some subgroups showing a significant reduction in risk for the ablation group. Specifically, females (HR 0.69, *p* = 0.0402), patients with LVEF ≥ 57% (HR 0.66, *p* = 0.0474), with HTN (HR 0.72, *p* = 0.0182), without DM (HR 0.70, *p* = 0.0367), and paroxysmal AF (HR 0.72, *p* = 0.0131). (C) Subgroup analysis of hazard ratios for HF with acute exacerbation with ablation versus without ablation. The HRs indicate a varying degree of risk for HF exacerbation across different subgroups, with some subgroups showing a significant reduction in risk for the ablation group. Specifically, females (HR 0.73, *p* = 0.0086), patients with LVEF ≥ 57% (HR 0.73, *p* = 0.0094), without CKD (HR 0.76, *p* = 0.0063), with HTN (HR 0.74, *p* = 0.0015). AF, atrial fibrillation; CI, confidence interval; CKD, chronic kidney disease; DM, diabetes mellitus; HF, heart failure; HR, hazard ratio; HTN, hypertension; LVEF, left ventricular ejection fraction.

A subgroup analysis for all‐cause mortality of various patient characteristics showed the comparison of patients with ablation versus without ablation (Figure 2). Subgroup analysis showed significantly reduced mortality for patients with ablation across all categories, including different age, sex, LVEF, CKD, HTN, DM, dyslipidemia, and different AF types (Figure 2A).

A subgroup analysis for ischemic stroke of various patient characteristics presented a comparison of patients with ablation versus without ablation (Figure 2B). The analysis showed ablation was associated with significantly lower HRs in females (HR 0.69, *p* = 0.0402), patients with LVEF ≥ 57% (HR 0.66, *p* = 0.0474), those without CKD (HR 0.74, *p* = 0.0235), those with HTN (HR 0.72, *p* = 0.0182), and those with paroxysmal AF (HR 0.72, *p* = 0.0131).

A subgroup analysis for HF with acute exacerbation of various patient characteristics showed a comparison of patients with ablation versus patients without ablation (Figure 2C). The analysis showed that ablation was associated with significantly lower HRs in females (HR 0.73, *p* = 0.0086), patients with LVEF ≥ 57% (HR 0.73, *p* = 0.0094), those without CKD (HR 0.76, *p* = 0.0063), patients with HTN (HR 0.74, *p* = 0.0015), and those with dyslipidemia (HR 0.72, *p* = 0.0012).

The subgroup analyses highlighted the heterogeneous impact of ablation therapy across different patient subgroups and indicated that the benefits of ablation may be more pronounced in specific populations.

## 4 Discussion

The benefits of CA for patients with HFpEF and AF remain a controversial issue, with current evidence being insufficient for a definitive conclusion. In this study, we observed a lower incidence of all‐cause mortality, stroke, and HF with acute exacerbation in patients with HFpEF and AF following CA. Additionally, there was a trend toward a lower incidence of progression to HFrEF after CA. The reduction in mortality was consistent across all subgroups, while the reduction in stroke and HF exacerbation was more significant in specific groups, particularly females, those with better LVEF, without CKD, with HTN, and in patients with paroxysmal AF. These findings suggest that ablation therapy's benefits may vary, being more pronounced in certain populations.

### 4.1 CA Provided the Benefit of Reducing HF Hospitalizations and May Slow the Progression to Reduced LVEF

In patients with AF and HFrEF, several randomized controlled trials and meta‐analyses have demonstrated the effectiveness of CA in improving survival, HF hospitalizations, functional capacity, and quality of life, with acceptable safety [2, 10, 11, 12, 13, 14]. In patients with LVEF < 50%, 75.0% showed improvement in LVEF following CA for AF [15]. Predictors of this improvement included a smaller left ventricular volume and the absence of low voltage zones in the left atrium [15, 16]. CA also slowed the progression of LA remodeling [7, 17]. However, the improvement of LVEF observed in some studies was not confirmed in patients with HFpEF following CA. One meta‐analysis reported that CA had limited or no benefit in reducing cardiovascular and all‐cause mortality for patients with AF and HFpEF [11]. However, another meta‐analysis showed different results from more studies, showing that CA reduced HF hospitalizations and increased the prevalence of maintaining sinus rhythm [18]. Additionally, another report showed various benefits of CA for patients with HFrEF and HF with mildly reduced EF, including improved LVEF, increased walking distance, reduced AF recurrence, and lower all‐cause mortality [19]. In our study, we found that the incidence of HF with acute exacerbation was significantly lower in patients who underwent CA compared to those who received medical treatment alone, especially in the subgroups of female, EF ≥ 57%, with HTN, and without CKD. These findings suggested that CA could still be beneficial in the management of HF by reducing the frequency of hospital admissions in patients with HFpEF in real‐world practice. Although CA showed potential in slowing the progression to reduced EF, the difference was not statistically significant. This finding indicated that while CA offered certain benefits in the management of HF in patients with HFpEF, its impact on LVEF progression requires further investigation.

### 4.2 The Impact of CA on Risk Reduction of Stroke in HFpEF and AF

Reducing the burden of arrhythmia is a reasonable strategy as it may help decrease the risk of stroke. In a randomized controlled study on early rhythm control of AF, a lower incidence of stroke was observed in the population receiving early rhythm control through medications or CA [3]. However, the benefit of rhythm control of AF by CA was not observed in studies for patients with HFrEF, although there may still be a trend toward a reduced risk of stroke [4]. CA could increase the rate of maintenance of sinus rhythm and improved quality of life in patients with AF and HFpEF [20, 21]. Additionally, one meta‐analysis concluded that CA significantly reduced the risk of stroke compared to medical therapy alone [22]. While the contribution of CA to stroke reduction in AF patients remains a controversial issue, there may be a trend toward its benefit in some studies [23, 24]. In this study of CA for AF in patients with HFpEF, we observed that the 1‐ and 5‐year stroke risks were significantly reduced in the ablation group compared to the non‐ablation group. This study showed that CA provided some benefit in reducing stroke events in specific population, especially in females, patients with an EF ≥ 57%, patients without CKD, patients with HTN, patients without type 2 DM, and patients with paroxysmal AF.

### 4.3 Study Limitations

The first limitation, inherent to all observational studies, is residual unmeasured confounding factors. Additionally, our data was obtained retrospectively from electronic medical records, which rely on the accurate documentation of diagnostic codes, introducing potential limitations due to miscoding. Another limitation is that the index date for the ablation cohort was the date of the CA, whereas for the non‐ablation cohort it was the date of the initial co‐diagnosis of AF and HFpEF. This difference means patients in the ablation group may have been captured at a later stage of their disease course, potentially introducing lead‐time or immortal‐time bias. Another limitation is the inability to determine and compare the duration of AF or HFpEF before the index date, as diagnostic codes may not reflect true disease onset. Consequently, differences in disease duration between groups could not be fully adjusted for. Another limitation is that we could not stratify by ablation technique (e.g., radiofrequency vs. cryoballoon) due to inconsistent capture of procedural details. Furthermore, the database only captures outcomes if a patient remains within the same or another participating HCO, which means some outcomes may have been missed. However, the bias should have affected in both study groups equally. Lastly, social determinants of health and other unmeasurable confounding factors may have influenced the outcomes, which we were unable to account for. Even though PSM was performed to reduce bias, there are still unmeasured confounding factors that may affect the results. These unmeasured factors could potentially influence the outcomes in ways that were not accounted for in the analysis, highlighting the inherent limitations of observational studies despite advanced statistical techniques like PSM.

## 5 Conclusions

In patients with AF and HFpEF, CA provided cardiovascular and cerebral benefits and might reduce the risk of progression to HFrEF over 5 years of follow‐up. Additionally, CA was associated with a reduction in all‐cause mortality in patients with AF and HFpEF.

## Consent

The IRB waived the requirement for written informed consent due to the retrospective nature of the study.

## Conflicts of Interest

The authors declare no conflicts of interest.

## Supporting information

Supplemental tables R1.

Supplemental Figure 1.

## Data Availability

Data from this study can be obtained from the corresponding authors upon reasonable request. This study utilized data from the TriNetX research network. The analyses, interpretations, and conclusions drawn in this study do not reflect the views of Chi Mei Medical Center.
