# Supplementary material for: Evaluating the Impact of Catheter Ablation on Cardiovascular and Cerebral Outcomes in Atrial Fibrillation With Heart Failure and Preserved Ejection Fraction
Source: Clin Cardiol. 2025 Nov 7;48(11):e70220. doi: 10.1002/clc.70220 (PMC12592939; doi:10.1002/clc.70220)
Supplement: Supplementary file 1 — Supplemental tables R1. [file CLC-48-e70220-s002.docx]

| **Supplemental Table 1.** Demographic, diagnostic, procedural, medication, visit, and laboratory codes utilized in the definition of the cohorts. | | |
| --- | --- | --- |
| **Category** | **Code** | **Description** |
| **#1 Age at least 18 years** | |  |
| demographics | Age | Age (at least 18 years) |
| **#2 Visited HCOs at least 3 since Feb 1, 2014** | | |
| (must have any of the following since Feb 1, 2024) | | |
| visit | TNX:Visit | Number of occurrences: greater than or equal to 3 instances |
| **#3 Received ablation procedure** | |  |
| (must have any of the following for the with ablation group) | | |
| (must NOT have any of the following for the without ablation group) | | |
| procedure | UMLS:SNOMED:233159005 | Ablation operation for arrhythmia |
| procedure | UMLS:CPT:93656 | Comprehensive electrophysiologic evaluation including transseptal catheterizations, insertion and repositioning of multiple electrode catheters with intracardiac catheter ablation of atrial fibrillation by pulmonary vein isolation, including intracardiac electrophysiologic 3-dimensional mapping, intracardiac echocardiography including imaging supervision and interpretation, induction or attempted induction of an arrhythmia including left or right atrial pacing/recording, right ventricular pacing/recording, and His bundle recording, when performed |
| procedure | UMLS:CPT:93657 | Additional linear or focal intracardiac catheter ablation of the left or right atrium for treatment of atrial fibrillation remaining after completion of pulmonary vein isolation (List separately in addition to code for primary procedure) |
| **#4 Diagnosed with atrial fibrillation within 1 year on or before the first instance of #3** | | |
| (must have any of the following) | |  |
| diagnosis | UMLS:ICD10CM:I48.0 | Paroxysmal atrial fibrillation |
| diagnosis | UMLS:ICD10CM:I48.2 | Chronic atrial fibrillation |
| diagnosis | UMLS:ICD10CM:I48.91 | Unspecified atrial fibrillation |
| diagnosis | UMLS:ICD10CM:I48.1 | Persistent atrial fibrillation |
| **#5 Diagnosed with heart failure with preserved ejection fraction within 1 year on or before the first instance of #3** | | |
| (must have all of the following) | |  |
| diagnosis | UMLS:ICD10CM:I50 | Heart failure |
| laboratory | TNX:FINDING:2003 | Left Ventricular Ejection Fraction (LVEF) (%) (at least 50.00 %) |
| ( must NOT have the following) | |  |
| laboratory | TNX:FINDING:2003 | Left Ventricular Ejection Fraction (LVEF) (%) (at most 49.00 %) |
| **#6 Received anticoagulant treatment within 1 year on or before the first instance of #3** | | |
| medication | NLM:RXNORM:1037042 | dabigatran etexilate |
| medication | NLM:RXNORM:1114195 | rivaroxaban |
| medication | NLM:RXNORM:1364430 | apixaban |
| medication | NLM:RXNORM:1599538 | edoxaban |
| medication | NLM:RXNORM:11289 | warfarin |
| medication | NLM:RXNORM:1546356 | dabigatran |
| **#7 Excluded patients who ever received heart valve replacement, diagnosed with CKD stage 5, ESRD, SVT, or atrial flutter** | | |
| ( must NOT have any of the following) | |  |
| diagnosis | UMLS:ICD10CM:N18.5 | Chronic kidney disease, stage 5 |
| diagnosis | UMLS:ICD10CM:N18.6 | End stage renal disease |
| diagnosis | UMLS:ICD10CM:I47.1 | Supraventricular tachycardia |
| diagnosis | UMLS:ICD10CM:I48.92 | Unspecified atrial flutter |
| diagnosis | UMLS:ICD10CM:I48.3 | Typical atrial flutter |
| diagnosis | UMLS:ICD10CM:I48.4 | Atypical atrial flutter |
| procedure | UMLS:CPT:33369 | Transcatheter aortic valve replacement (TAVR/TAVI) with prosthetic valve; cardiopulmonary bypass support with central arterial and venous cannulation (eg, aorta, right atrium, pulmonary artery) (List separately in addition to code for primary procedure) |
| procedure | UMLS:SNOMED:232740008 | Mitral valve operation |
| procedure | UMLS:CPT:1035167 | Replacement, aortic valve |
| procedure | UMLS:CPT:33367 | Transcatheter aortic valve replacement (TAVR/TAVI) with prosthetic valve; cardiopulmonary bypass support with percutaneous peripheral arterial and venous cannulation (eg, femoral vessels) (List separately in addition to code for primary procedure) |
| procedure | UMLS:CPT:33364 | Transcatheter aortic valve replacement (TAVR/TAVI) with prosthetic valve; open iliac artery approach |
| procedure | UMLS:CPT:33361 | Transcatheter aortic valve replacement (TAVR/TAVI) with prosthetic valve; percutaneous femoral artery approach |
| procedure | UMLS:CPT:33363 | Transcatheter aortic valve replacement (TAVR/TAVI) with prosthetic valve; open axillary artery approach |
| procedure | UMLS:CPT:33362 | Transcatheter aortic valve replacement (TAVR/TAVI) with prosthetic valve; open femoral artery approach |
| procedure | UMLS:CPT:33366 | Transcatheter aortic valve replacement (TAVR/TAVI) with prosthetic valve; transapical exposure (eg, left thoracotomy) |
| procedure | UMLS:CPT:33368 | Transcatheter aortic valve replacement (TAVR/TAVI) with prosthetic valve; cardiopulmonary bypass support with open peripheral arterial and venous cannulation (eg, femoral, iliac, axillary vessels) (List separately in addition to code for primary procedure) |
| procedure | UMLS:CPT:33365 | Transcatheter aortic valve replacement (TAVR/TAVI) with prosthetic valve; transaortic approach (eg, median sternotomy, mediastinotomy) |
| procedure | UMLS:CPT:1021150 | Transcatheter aortic valve replacement (TAVR/TAVI) with prosthetic valve |
| procedure | UMLS:SNOMED:232846007 | Aortic valve replacement and replacement of ascending aorta |
| diagnosis | UMLS:ICD10CM:Z95.3 | Presence of xenogenic heart valve |
| procedure | UMLS:SNOMED:725351001 | Transcatheter aortic valve replacement (deprecated 2022) |
| procedure | UMLS:SNOMED:232848008 | Aortic valve replacement and patch aortoplasty |
| diagnosis | UMLS:ICD10CM:Z95.2 | Presence of prosthetic heart valve |
| procedure | UMLS:SNOMED:174928005 | Xenograft aortic valve replacement |
| procedure | UMLS:SNOMED:174927000 | Homograft aortic valve replacement |
| procedure | UMLS:SNOMED:174929002 | Mechanical prosthetic aortic valve replacement |
| procedure | UMLS:SNOMED:308663002 | Porcine aortic valve replacement |
| procedure | UMLS:SNOMED:232847003 | Aortic valve replacement and aortoplasty |
| procedure | UMLS:SNOMED:275199000 | Ball valve aortic valve replacement |
| procedure | UMLS:SNOMED:34068001 | Heart valve replacement |

| **Supplemental Table 2.** Demographic, diagnostic, and laboratory codes utilized in the definition of covariates. | | | |
| --- | --- | --- | --- |
| **Category** | **Code** | **Description** |  |
| Demographics | AI | Age at Index |  |
| Demographics | F | Female |  |
| Demographics | 56370 | Black or African American |  |
| Demographics | M | Male |  |
| Demographics | 75301 | White |  |
| Demographics | 1002-5 | American Indian or Alaska Native |  |
| Demographics | UNK | Unknown Race |  |
| Demographics | 64498 | Native Hawaiian or Other Pacific Islander |  |
| Demographics | UN | Unknown Ethnicity |  |
| Demographics | 104582 | Not Hispanic or Latino |  |
| Demographics | 85865 | Hispanic or Latino |  |
| Demographics | 84373 | Other Race |  |
| Demographics | 46997 | Asian |  |
| Diagnosis | I10-I1A | Hypertensive diseases |  |
| Diagnosis | I10-I15 | Hypertensive diseases (deprecated 2018) |  |
| Diagnosis | E78 | Disorders of lipoprotein metabolism and other lipidemias |  |
| Diagnosis | I20-I25 | Ischemic heart diseases |  |
| Diagnosis | E11 | Type 2 diabetes mellitus |  |
| Diagnosis | E66 | Overweight and obesity |  |
| Diagnosis | E00-E07 | Disorders of thyroid gland |  |
| Diagnosis | N18.3 | Chronic kidney disease, stage 3 (moderate) |  |
| Diagnosis | N18.4 | Chronic kidney disease, stage 4 (severe) |  |
| Diagnosis | N18.5 | Chronic kidney disease, stage 5 |  |
| Diagnosis | N18.6 | End stage renal disease |  |
| Diagnosis | I63 | Cerebral infarction |  |
| Diagnosis | I48.0 | Paroxysmal atrial fibrillation |  |
| Diagnosis | I48.1 | Persistent atrial fibrillation |  |
| Diagnosis | I48.2 | Chronic atrial fibrillation |  |
| Diagnosis | I48.91 | Unspecified atrial fibrillation |  |
| Diagnosis | I50.31 | Acute diastolic (congestive) heart failure |  |
| Medication | CV700 | DIURETICS |  |
| Medication | CV800 | ACE INHIBITORS |  |
| Medication | CV805 | ANGIOTENSIN II INHIBITOR |  |
| Medication | 1656328 | sacubitril |  |
| Medication | CV050 | DIGITALIS GLYCOSIDES |  |
| Medication | CV100 | BETA BLOCKERS/RELATED |  |
| Medication | CV200 | CALCIUM CHANNEL BLOCKERS |  |
| Medication | CV300 | ANTIARRHYTHMICS |  |
| Medication | C10AA | HMG CoA reductase inhibitors |  |
| Medication | HS501 | INSULIN |  |
| Medication | 6809 | metformin |  |
| Medication | A10BK | Sodium-glucose co-transporter 2 (SGLT2) inhibitors |  |
| Medication | 4821 | glipizide |  |
| Medication | BL117 | PLATELET AGGREGATION INHIBITORS |  |
| Medication | BL110 | ANTICOAGULANTS |  |
| Laboratory | 9024 | Creatinine [Mass/volume] in Serum, Plasma or Blood |  |
| Laboratory | 9002 | Cholesterol in LDL [Mass/volume] in Serum or Plasma |  |
| Laboratory | 9037 | Hemoglobin A1c/Hemoglobin.total in Blood |  |
| Laboratory | 9003 | Natriuretic peptide B [Mass/volume] in Serum, Plasma or Blood |  |
| Laboratory | 9072 | Natriuretic peptide.B prohormone N-Terminal [Mass/volume] in Serum, Plasma or Blood |  |
| Laboratory | 2003 | Left Ventricular Ejection Fraction (LVEF) (%) |  |

| **Supplemental Table 3.** Diagnostic, demographic, medication and laboratory codes utilized in the definition of outcomes. | | |
| --- | --- | --- |
| Index date: the first date of receiving ablation therapy for with ablation group, the first date of diagnosis of atrial fibrillation and HFpEF for without ablation group | | |
| Outcome time window: 3 months to 5 years after the index date | | |
| **Category** | **Code** | **Description** |
| **#1 All-cause mortality** | |  |
| (excluding patients with outcome prior to the time window) | | |
| Demographics | Deceased | Deceased |
| **#2 Heart failure with acute exacerbation** | | |
| Diagnosis | UMLS:ICD10CM:I50.21 | Acute systolic (congestive) heart failure |
| Diagnosis | UMLS:ICD10CM:I50.23 | Acute on chronic systolic (congestive) heart failure |
| Diagnosis | UMLS:ICD10CM:I50.41 | Acute combined systolic (congestive) and diastolic (congestive) heart failure |
| Diagnosis | UMLS:ICD10CM:I50.43 | Acute on chronic combined systolic (congestive) and diastolic (congestive) heart failure |
| Diagnosis | UMLS:ICD10CM:I50.31 | Acute diastolic (congestive) heart failure |
| Diagnosis | UMLS:ICD10CM:I50.33 | Acute on chronic diastolic (congestive) heart failure |
| Diagnosis | UMLS:ICD10CM:I50.811 | Acute right heart failure |
| Diagnosis | UMLS:ICD10CM:I50.813 | Acute on chronic right heart failure |
| Medication | NLM:VA:CV702 | LOOP DIURETICS (Route: Injectable Product) |
| **#3 LVEF progress to mildly reduced EF** | | |
| Laboratory | TNX:FINDING:2003 | Left Ventricular Ejection Fraction (LVEF) (%) (between 40.00 and 50.00 % (most recent occurrence)) |
| **#4 LVEF progress to reduced EF** | |  |
| Laboratory | TNX:FINDING:2003 | Left Ventricular Ejection Fraction (LVEF) (%) (at most 40.00 % (most recent occurrence)) |
| **#5 Cerebral infarction** | |  |
| Diagnosis | UMLS:ICD10CM:I63 | Cerebral infarction |

| **Supplemental Table 4.** Demographic, diagnostic, procedural, and medication codes utilized in the definition of the subgroup analysis | | |
| --- | --- | --- |
| **#1 Age at index** |  |  |
| **#1.1 Age <75** |  |  |
| demographics | Age | between 18 and 74 years |
| **#1.2 Age ≥ 75** |  |  |
| demographics | Age | at least ≥ 75 years |
| **#2 Sex** |  |  |
| **#2.1 Female** |  |  |
| demographics | UMLS:HL7V3.0:Gender:F | Female |
| **#2.2 Male** |  |  |
| demographics | UMLS:HL7V3.0:Gender:M | Male |
| **#3 Baseline LVEF** | |  |
| **#3.1 LVEF < 57%** | |  |
| (must have any of following prior to the index date) | | |
| laboratory | TNX:FINDING:2003 | Left Ventricular Ejection Fraction (LVEF) (%) (between 50.00 and 56.99 % (most recent occurrence)) |
| **#3.1 LVEF ≥ 57%** | |  |
| (must have any of following prior to the index date) | | |
| laboratory | TNX:FINDING:2003 | Left Ventricular Ejection Fraction (LVEF) (%) (at least 57.00 % (most recent occurrence)) |
| **#4 Chronic kidney disease (CKD)** | |  |
| #4.1 With CKD |  |  |
| (must have any of following prior to the index date) | | |
| diagnosis | UMLS:ICD10CM:N18.3 | Chronic kidney disease, srage 3 (moderate) |
| diagnosis | UMLS:ICD10CM:N18.4 | Chronic kidney disease, srage 4 (severe) |
| #4.2 Without CKD | |  |
| (must NOT have any of #4.1 prior to the index date) | | |
| **#5 Hypertension (HTN)** | |  |
| #5.1 With HTN |  |  |
| (must have any of following prior to the index date) | | |
| diagnosis | UMLS:ICD10CM:I10-I1A | Hypertensive diseases |
| diagnosis | UMLS:ICD10CM:I10-I15 | Hypertensive diseases (deprecated 2018) |
| #5.2 Without HTN | |  |
| (must NOT have any of #5.1 prior to the index date) | | |
| **#6 Type 2 diabetes mellitus (T2DM)** | |  |
| #6.1 With T2DM |  |  |
| (must have the following prior to the index date) | | |
| diagnosis | UMLS:ICD10CM:E11 | Type 2 diabetes mellitus |
| #6.2 Without T2DM | |  |
| (must NOT have #6.1 prior to the index date) | | |
| **#7 Dyslipidemia** |  |  |
| #7.1 With dyslipidemia | |  |
| (must have the following prior to the index date) | | |
| diagnosis | UMLS:ICD10CM:E78 | Disorders of lipoprotein metabolism and other lipidemias |
| #7.2 Without dyslipidemia | |  |
| (must NOT have #7.1 prior to the index date) | | |
| **#8 Types of atrial fibrillation** | |  |
| #8.1 Paroxysmal atrial fibrillation | |  |
| (must have the following prior to the index date) | | |
| diagnosis | UMLS:ICD10CM:I48.0 | Paroxysmal atrial fibrillation |
| #8.2 Non-paroxysmal atrial fibrillation | |  |
| (must NOT have #8.1 prior to the index date) | | |

**Supplemental Table 5** – Post Hoc Power Calculations for Estimated Hazard Ratios

| Outcomes | Power (%)^1^ |
| --- | --- |
| All-cause mortality | >99.9 |
| HF exacerbation | >99.9 |
| LVEF progress to mildly reduced EF | 4.5 |
| LVEF progress to reduced EF | 96.4 |
| Ischemic stroke | >99.9 |

^1^ Power is calculated using the Schoenfeld method, based on the observed number of events and effect sizes, with a two-sided alpha level of 0.05. Outcomes with power ≥80% were considered adequately powered to detect the observed effect sizes.

Abbreviation: HF: heart failure; LVEF: left ventricular ejection fraction.

**Supplemental Figure legends**

Figure 1. Flowchart of study population selection and propensity score matching

This flowchart illustrated the selection process of the study population from the Global Collaborative Network in TriNetX, which includes 120 healthcare organizations (HCOs) worldwide. Data were retrieved on June 24, 2024, encompassing a total of 128,873,996 individuals. From this dataset, patients aged 18 years or older who had visited HCOs at least three times since February 1, 2014, were identified (n = 65,160,687). Among these, patients diagnosed with heart failure with preserved ejection fraction (HFpEF) and atrial fibrillation were selected (n = 32,024).

The following exclusion criteria were applied:

- Patients who did not receive anticoagulant treatment (n = 14,284)
- Patients who had ever received heart valve replacement (n = 753)
- Patients diagnosed with chronic kidney disease (CKD) stage 5 or end-stage renal disease (ESRD) (n = 894)
- Patients diagnosed with supraventricular tachycardia (SVT) or atrial flutter alone (n = 2,020)

After applying these criteria, patients were divided into two groups: those who received ablation therapy (n = 1,234) and those who did not receive ablation therapy (n = 13,500). Propensity score matching (1:1) was performed by age, gender, race, ethnicity, comorbid conditions, medications, and laboratory features, resulting in 1,152 patients in each cohort. The index date was defined as the first date of receiving ablation for the ablation group and as the first date of diagnosis of AF with HFpEF for the without ablation group.

Abbreviation: HCOs: healthcare organizations; HFpEF: heart failure with preserved ejection fraction; CKD: chronic kidney disease; ESRD: end-stage renal disease; SVT: supraventricular tachycardia.
