# Supplementary figures and images for: Evaluating the Impact of Catheter Ablation on Cardiovascular and Cerebral Outcomes in Atrial Fibrillation With Heart Failure and Preserved Ejection Fraction
Source: Clin Cardiol. 2025 Nov 7;48(11):e70220. doi: 10.1002/clc.70220 (PMC12592939; doi:10.1002/clc.70220)

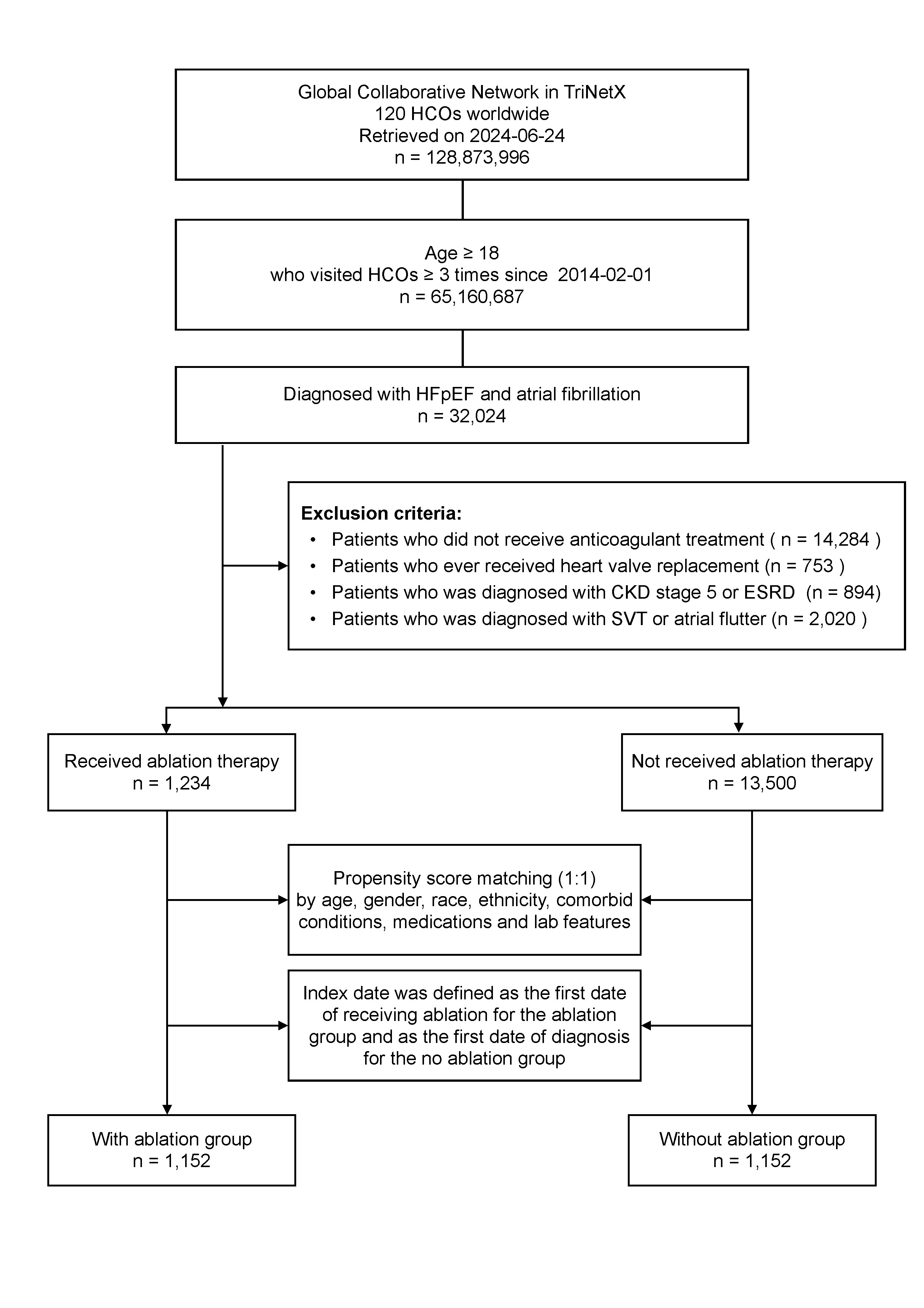

Supplement: Supplementary file 2 — Supplemental Figure 1. [file CLC-48-e70220-s001.jpg]
